# Evaluation of Vamana and Virechana as Interventions to Decelerate the Aging Process in Healthy Adult Volunteers: Protocol for an Exploratory Single-Arm Clinical Trial

**DOI:** 10.2196/99146

**Published:** 2026-09-16

**Authors:** Devipriya Soman, Ramesh NV, Pravith NK

**Affiliations:** 1 Department of Kayachikitsa, School of Ayurveda, Amrita Vishwa Vidyapeetham, Amritapuri, Kerala, 690525, India, 91 9495638076; 2 Department of Rasashastra and Bhaishajyakalpana, School of Ayurveda, Amrita Vishwa Vidyapeetham, Amritapuri, Kerala, India; 3 Department of Kayachikitsa, Government Ayurveda College, Thripunithura, Kerala, India

**Keywords:** Ayurveda, Vamana, Virechana, aging, study protocol

## Abstract

**Background:**

*Vamana* (therapeutic emesis) and *Virechana* (therapeutic purgation) are the two major biopurification procedures enlisted in Ayurveda. Studies explicitly showcasing their clinical efficacy are documented in substantial numbers. *Ritu sodhana* (seasonal purification) imparts rejuvenation in healthy individuals. However, its efficacy for health span and decelerating the aging process in healthy individuals remains a gray area.

**Objective:**

This study’s objective is to explore the effect of *ritu sodhana* on modifying serum levels of malondialdehyde (MDA), total superoxide dismutase (T-SOD), and telomerase activity.

**Methods:**

The study was prospectively registered in the Clinical Trial Registry-India (CTRI; CTRI/2024/02/062176), uses a pre-posttest study design, and includes 53 participants 35 years to 65 years old. The study is exploratory and lacks a control group. Those with a BMI between 18.5 kg/m^2^ and 25 kg/m^2^, with stable weight for 3 months immediately prior to the study, with *madhyama koshta* (medium nature of bowel evacuation, as assessed using aquestionnaire), who are *Vamanarha* (eligible for therapeutic emesis) and *Virechanarha* (eligible for therapeutic purgation), and who do not meet the exclusion criteria will be included in the study. The interventions include emesis in the spring and purgation in the autumn season, preceded by *Snehana* (oleation) and *Swedana* (fomentation) and followed by the *Samsarjana karma* (specialized menu of diets) depending on the measurement of purification. Trial duration will range from 18 days to 20 days for each participant, and the total study duration will be 3 years. The serum levels of MDA, SOD, and telomerase activity will be measured on day 0 and on the next day following completion of the *Samsarjana karma* (18th-20th day). Continuous variables for primary biological endpoints including serum MDA, T-SOD, and telomerase activity will be evaluated using paired-samples *t* tests or Wilcoxon signed-rank tests, with potential missing data managed under a missing completely at random assumption using available case analysis (pairwise deletion) cross-verified by a conservative baseline observation carried forward (BOCF) sensitivity test. The effect size will be also determined.

**Results:**

Recruitment began in March 2025. Data for 27 participants who voluntarily participated in *Vasantha Vamana* were collected in March 2025 and April 2025. Data analysis is pending, and the study is expected to be completed by the end of December 2026.

**Conclusions:**

The study will provide hypothesis-generating evidence, and the data will serve as a necessary precursor to adequately powered, confirmatory trials on the aging process.

## Introduction

### Background

*Vamana* (therapeutic emesis) and *Virechana* (therapeutic purgation) are procedures that help eliminate the accumulated morbid *dosha* (humor) from the body. They are considered the 2 major types of *sodhana* (biopurification) [1]. It has been suggested that “*chirat cha pakam vayasah karoti, samsodhanam samyak upasyamanam*” (ie, body purification delays aging) [2]. Another text says, “*jaram kruchrena labhate chiram jeevatyanamaya*”—a properly administered *sodhana* is able to arrest aging and provide an extended and healthy life span [3].

Retarding the aging process has been considered a target for slowing degenerative diseases. It is also considered a treatment option for tumor suppression. Although the average life expectancy has improved in the last 10 decades, there is no equivalent improvement in healthy life expectancy, which has been termed health span. Data published by the World Health Organization (WHO) in 2019 showed that life expectancy in India was 70.8 years, whereas the health life expectancy was only 60.3 years [4]. Hence, a number of cellular-level and experimental studies are being conducted in this area of research [5,6]. Clinical studies involving humans number the fewest in this area [7].

In a study carried out with 30 healthy volunteers, there was a significant increase in superoxide dismutase (SOD) levels and decrease in malondialdehyde (MDA) levels in the groups administered one 500-mg capsule of *Ashwagandha* and *Guduchi* twice a day for 6 months. The study concluded that *Ashwagandha* and *Guduchi* may be helpful for preventing oxidative stress and premature aging [8]. The results of another study indicated an increase in telomerase activity with no discernible change in telomere length in healthy volunteers aged 45 years to 60 years administered *Amalaki* for 45 days after the *koshta shuddhi* procedure. Telomerase activity and telomere length were analyzed in peripheral blood mononuclear cells on days 0, 45, and 90 of *Amalaki Rasayana* administration. The study concluded that maintenance of telomere length is facilitated by an increase in telomerase activity upon Rasayana administration in older adults and *Amalaki Rasayana* may prevent the erosion of telomeres over a period of time to promote healthy aging in older adults [9]. The randomized controlled trial by Dandekar and Kuchewar [10] investigated the effects of *Vasantik Vamana* karma (therapeutic emesis during spring) on oxidative stress. It found that the procedure significantly reduced MDA levels and increased SOD activity in healthy volunteers. A review on telomerase activators commmeted upon the limited literature on Ayurvedic telomerase activators [11]. The study conducted by Raghuraman and Subramaniam [12] reported a dose-dependent increase in telomerase activity up to 50 μg/mL; after administration, the extract successfully boosted telomerase activity up to a certain dose (50 μg/ml), but adding more than that caused the mixture to become toxic, lowering the cells’ survival rate because the extract did not dissolve properly in the liquid. DLBS1649, a novel extract derived from *Mandukaparni*, was evaluated in lifespan extension studies in fruit flies (*Drosophila*) and showed significant increase in mean survival time in male (>20%) and female (>10%) flies [13].

*Sodhana* can be practiced even by a healthy individual [14]. *Sodhana* offers stability to all bodily tissues, corrects the process of digestion, imparts strength and immunity, favors spermatogenesis and oogenesis, offers relief from diseases, returns an individual to normal physiology, aids with precise sensory perception, enhances mental status, clears the complexion, delays aging, and enables a healthy life span [15]. All these outcomes of *sodhana* are directly opposite to the factors that accelerate the aging process, as per Ayurveda principles. Hence, this study is being conducted to explore the potential of *Vamana* and *Virechana* for decelerating the aging process in healthy volunteers.

### Study Objectives

#### Primary Objective

The primary objective is to evaluate *Vamana* in *Vasanta ritu* (spring season) and *Virechana* in *Sharad ritu* (autumn season) for modulating systemic oxidative stress by measuring the reduction in serum levels of MDA and enhancement of SOD activity from baseline to postintervention.

#### Secondary Objective

The secondary objective is to determine the change in serum telomerase activity from baseline to postintervention.

## Methods

### Trial Design

This open-label, exploratory clinical trial consists of a single group and a primary endpoint of decreasing serum MDA values and increasing serum SOD values within 18 days to 20 days after *Vamana* in *Vasantha ritu* and *Virechana* in *Sarad ritu*. The participants will be recruited by the principal investigator consecutively by season: *Vamana* cohort in the *Vasantha ritu* (spring season) and *Virechana* cohort in the *Sarad ritu* (autumn season). Each participant is enrolled into only one seasonal arm based strictly on their chronological window of voluntary registration. There is no active assignment of participants; instead allocation follows a participant-driven, self-selection framework. The total study duration is 3 years. The study is registered in the Clinical Trial Registry-India (CTRI; CTRI/2024/02/062176).

### Trial Estimand

#### Overview

In accordance with the International Council on Harmonisation (ICH) E9(R1) statistical guidelines, the primary treatment effect targeted for evaluation is precisely defined through a single primary estimand structured across the target population, alternative treatment conditions, primary variables (endpoints), population-level summary measure, and handling of intercurrent events.

#### Target Population

The population consists of healthy adult volunteers aged 35 years to 50 years, residing in Kerala, India, who have been cleared of any baseline noncommunicable diseases (NCDs) or metabolic comorbidities via our 3-tier screening matrix.

#### Alternative Treatment Conditions

This includes the intra-individual pre-intervention baseline status (T₀) compared against the postintervention state (T₁) following the completion of a season-specific, inpatient *Śodhana* (*Vamana* or *Virechana*) and graduated *Samsarjana Krama* package.

#### Primary Variables (Endpoints)

The endpoints consist of continuous laboratory biomarker change scores measuring oxidative stress indicators, specifically MDA and SOD, and cellular longevity parameters, including telomerase activity.

#### Population-Level Summary Measure

The mean intra-individual change score (mean delta difference) will be calculated from baseline to postintervention across the pooled cohort of 53 participants.

#### Handling of Intercurrent Events

Intercurrent events anticipated in this protocol include clinical dropout, voluntary withdrawal, or protocol nonadherence (such as violating the inpatient *Ahara* or *Vihara* constraints). To handle these events, a “While-On-Treatment” strategy will be used. The target estimation focuses strictly on the biological treatment effect achieved while participants successfully adhere to the intensive inpatient intervention. Data points collected up to the exact moment of any protocol violation or withdrawal will be retained, while missing postevent endpoints will be mathematically handled using multiple imputation methods within our multivariable regression models, preventing bias from complete-case attrition.

### Study Setting

**Figure 1.**
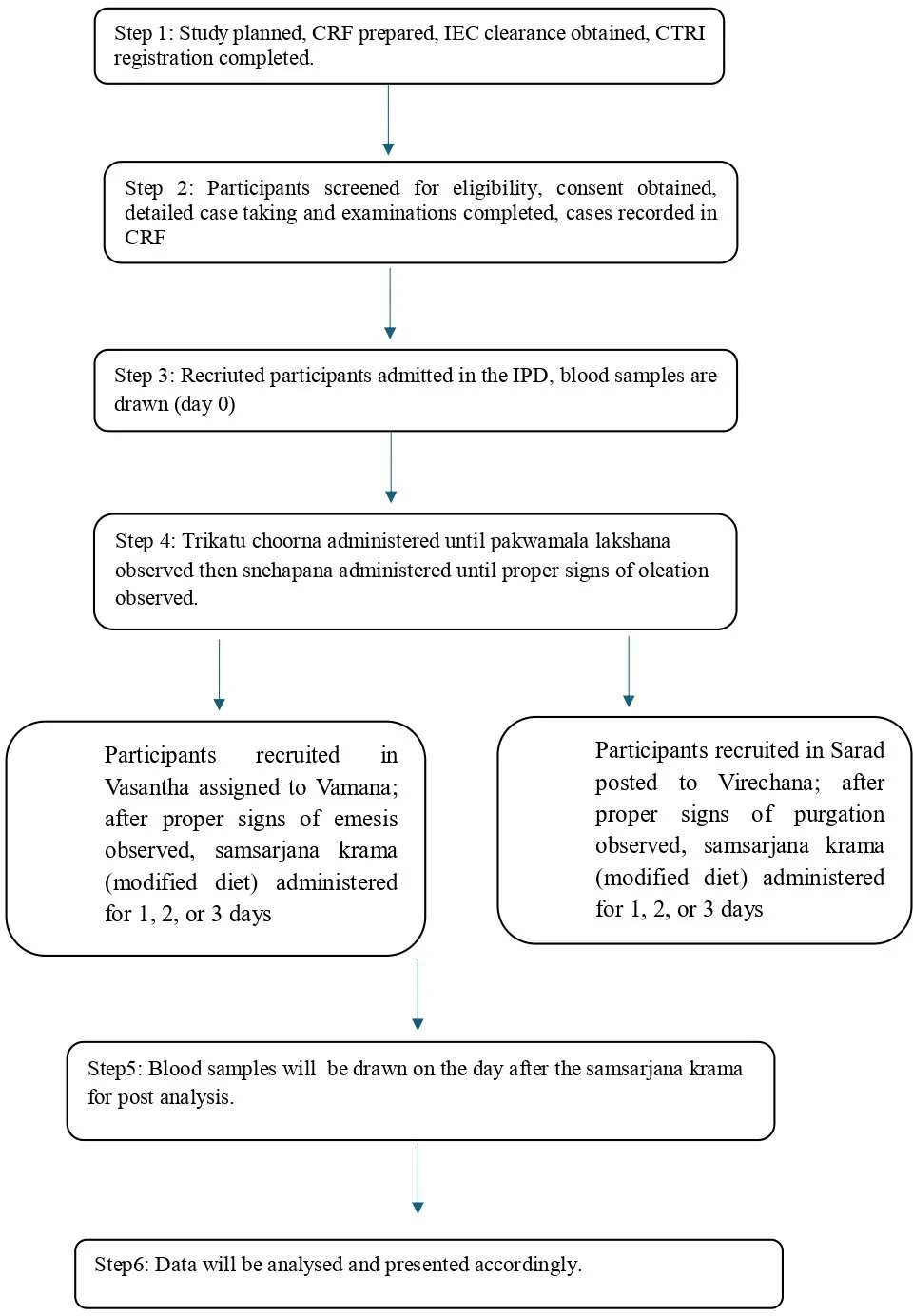
Summary of the methodology. CRF: case report form; CTRI: Clinical Trial Registry-India; IEC: institutional ethics committee; IPD: inpatient department.

The study is conducted at Amrita School of Ayurveda, Kollam, Kerala, and recruits participants from India only. The institute is a constituent of Amrita Vishwa Vidyapeetham and supports teaching and research. Figure 1 depicts the flowchart of the trial’s methodology.

### Primary Purpose

The intervention aims to decelerate the process of aging in humans. The date of first enrollment was March 2025, and the target sample size was 53 healthy volunteers. This trial is undertaken as part of a doctoral research project. The trial is ongoing and still recruiting participants. The total trial duration is 3 years.

### Eligibility Criteria

#### Inclusion Criteria

Healthy volunteers of either gender are included if they are aged 35 years to 60 years; are registered in the outpatient department, School of Ayurveda, Amrita Vishwa Vidyapeetham, Amritapuri, India; are willing to take part in the study; have a BMI between 18.5 kg/m^2^ and 25 kg/m^2^, have a stable weight (change <±10%) for 3 months immediately prior to the study; have *madhyama koshta* (medium nature of bowel evacuation, as assessed using a questionnaire); and are *vamanarha* (eligible for therapeutic emesis) and *virechanarha* (eligible for therapeutic purgation).

#### Exclusion Criteria

Indviduals were excluded for the following reasons: pregnancy or currently breastfeeding; addicted and unwilling to abstain from tobacco, alcohol, or recreational drugs; history of eating disorders; history of an acute or chronic inflammatory disorder; use of antidepressants or hormone replacement therapy; current diagnosis of posttraumatic stress disorder; unwilling to abstain from rich sources of resveratrol such as antioxidants including vitamin supplements, daily vitamins, resveratrol, minerals, dry grapes, blue berry, peanut butter, dark chocolate, and soy for the duration of study; unwilling to eat only study-provided food during the preconditioning and intervention periods; unstable weight (change >–10%) in the 3 months immediately prior to the study; and food allergies and dietary restrictions.

Those who are unwilling to eat only study-provided food during pre-conditioning and intervention periods.

### Evaluation of Healthy Volunteers

All the participants will be evaluated for healthy status through a clinical assessment and baseline laboratory screening.

The clinical assessment consists of a complete history-taking including usage of any current and concomittant medication, and a thorough systemic clinical examination including monitoring of vital signs will be performed at baseline. Any clinical findings deviating from the defined status of healthy volunteers will result in exclusion of the candidate.

Baseline laboratory screening includes a complete blood count (CBC) with erythrocyte sedimentation rate, lipid profile, blood glucose levels, renal function test, liver function test, thyroid function test, and urine routine examination to rule out any existing co-morbidity. The results of all these tests must fall within the standard physiological reference range for a participant to be includeds in the study.

### Strategies for Enrollment Promotion and Recruitment Feasibility

This research study is directly embedded within a long-standing, highly popular seasonal clinical service at our institute. Hence, external promotional advertisements, public campaigns, or medical record prescreening are not required to achieve our target sample size. The institute possesses an organic, highly motivated pool of community members who voluntarily register for the *Vasantha Vamana* and *Sharad Virechana* health maintenance sessions on their own initiative each year. To promote adequate enrollment and successfully secure our cohort of 53 healthy participants from this existing pipeline, our strategy focuses on systematic opportunistic screening and minimizing volunteer burden.

### Interventions

#### *Vamana* (Therapeutic Emesis)

##### *Poorvakarma* (Preoperative Procedure)

*Pachana* (carminative)–*Deepana* (digestive) with *Trikatu choorna* (herbal powder containing *Terminalia chebula, Terminalia bellarica,* and *Embelica officinalis*) is administered until pakwa mala lakshanas (symptoms indicative of proper digestion) are observed.

Recruited participants receive 3 gm *Trikatu choorna* with one-half glass of water twice daily after food for a period of 5 days to 7 days.

*Snehapana* (oleation) with *Moorchita ghruta* (ghee processed with herbs) is administered until *samyak snigdha lakshana* (symptoms indicative of proper oleation) are observed (using a validated tool).

Following the observation of *pakwa mala lakshana*, the participants will be advised to drink ghee. The ghee will be administered in increasing doses (depending on digestive capacity) starting at 30 mL. The time of administration will be around 7 AM. Upon consuming the ghee, the participant should sip warm water and wait until the ghee is digested properly (observed by the appearance of hunger and clear belching devoid of taste of the ghee). The same process is repeated for 5 days to 7 days.

For *Kaphotkleshakara Ahara*, once we observe the proper signs of oleation, idli (a South Indian preparation) with sugar and milk (breakfast), rice with curd (lunch), *tila* laddu (sesame balls)/*masha* (sweet pudding made of blackgram) *payasam* (evening snack), and rice and milk (dinner), all in quantities per the digestive capacity of the individual, will be administered on the next day.

*Sarvanga abhyanga* (whole body massage) with *moorchita taila* (oil processed with herbs) followed by *Bashpa sweda* (fomentation) will also be performed on the same day.

##### *Pradhana Karma* (Main Procedure)

*Vamana* will be conducted using *Madanaphala yoga* (drug prepared from *Randia dumetorum*) on the next day preceded by *Sarvanga abhyanga* (whole body massage) with *moorchita taila* followed by *Bashpa sweda* (fomentation) in the morning around 6:30 AM.

For *paschat karma* (the postoperative procedure), *Gandusha* (gargling), *Dhoomapana* (inhaling medicated fumes), and washing the lower part of the body with warm water will be performed.

*Samsarjana krama* (modified dietic regimen) will then be advised based on *sudhi lakshana* (signs and symptoms suggestive of a proper therapeutic emesis) for 1, 2, or 3 days.

### *Virechana* (Therapeutic Purgation)

#### *Poorvakarma* (Preoperative Procedure)

*Pachana* (carminative)–*Deepana* (digestive) with *Trikatu choorna* is administered until *pakwa mala lakshana* are observed.

Recruited participants receive 3 gm of *Trikatu choorna* with one-half glass of water twice daily after food for a period of 5 days to 7 days.

*Snehapana* (oleation) with *Moorchita ghruta* is administered until *samyak snigdha lakshana* are observed (using a validated tool).

After observation of *pakwa mala lakshana*, the participants will be advised to drink ghee. The ghee will be administered in increasing doses (depending on digestive capacity) starting at 30 mL. The time of administration will be around 7 AM. Upon consuming the ghee, the participant should sip warm water and wait until the ghee is digested properly (observed by appearance of hunger and clear belching devoid of the taste of the ghee). This process is repeated for 5 days to 7 days.

*Sarvanga abhyanga* (whole body massage) with *Moorchita taila* followed by *Bashpa sweda* (fomentation) will be administered for 2 days prior to virechana and on the day of *virechana*.

#### *Pradhana Karma* (Main Procedure)

*Virechana* will be completed using *Trivrit Lehya* (drug prepared from *Operculina turperthum*) at around 7 AM.

For *paschat karma* (the postoperative procedure), after attaining hunger and when the participant ceases to feel the urge for defecation, he/she will be allowed to take a bath in warm water.

Based on the *sudhi lakshana* (signs and symptoms suggestive of a proper purgation), the participant will be advised to follow *samsarjana* (modified dietic regimen) for 1, 2, or 3 days.

The participants will be admitted to the hospital for the duration of the trial. The administered diet will be uniform for all participants. Hydration will also be monitored. Participants are restrained from doing strenuous physical activity throughout the intervention. They are also advised to sleep for a strict 6 hours to 8 hours. The strictly enforced controls on diet, hydration, physical exertion, and sleep are not external protocol restrictions but standardized, fundamental therapeutic components (*Ahara* and *Vihara*) inherent to the classical *Śodhana* (Panchakarma) and *Samsarjana Krama* (graduated postdetox dietary timeline) intervention package. These parameters are operationally deployed within an institutional setting to ensure uniform clinical delivery.

### Intervention Provider Qualifications and Quality Control

The trial will be monitored and supervised by the principal investigator, who holds a Doctor of Medicine (MD) in Kayachikitsa (Ayurvedic Internal Medicine) and possesses 10 years of postgraduate clinical, research, and academic experience. The *Poorvakarma* procedures (including *Abhyanga* and *Sweda*) will be performed by following the institutional standard operating procedures (SOPs) by institutional Panchakarma therapists formally trained and employed at our institute. To ensure intervention fidelity and minimize practitioner-induced variability, all therapists will complete a mandatory, protocol-specific practical alignment session led by the principal investigator. Moreover, the investigator will audit the treatment sessions to ensure strict compliance with the protocol. As this is an exploratory study, a secondary objective is to evaluate the feasibility of the protocol. Intervention fidelity will be maintained through adherence to written institutional SOPs overseen by the principal investigator.

### Participant Adherence

The study is conducted under direct observation of the investigators. The participants will remain inpatients in the hospital (study setting). Every dose of *Snehapana*, the medicines to induce *Vamana* and *Virechana*, and all rescue medications, if any, will be administered directly under the visual supervision of the institutional nursing staff and verified by the investigator. Hence, for this trial, compliance shall be defined as 100% doses taken, excluding circumstances that will result in withdrawal of participants. Participant adherence rates will be descriptively tracked daily by the team to evaluate the tolerability of the purification phases. This tracking includes monitoring compliance with the daily titrated *Snehapana* volumes, *sodhna* procedure, and the *Samsarjana Krama*. The collected adherence data will directly inform the feasibility design and sample size calculations of future definitive trials.

To address the inherent adherence challenges of complex interventions like *Vamana* therapy, participant retention will be secured through structured preprocedural patient education and continuous clinical oversight. Prior to the procedure, participants will receive thorough education regarding the operational steps and safety parameters of *Vamana* to manage expectations and reduce procedural anxiety. Furthermore, the entire therapy will be executed under close clinical supervision; the principal investigator and trained nursing staff will remain at the bedside to monitor vitals, provide reassurance, and ensure strict protocol compliance.

### Rescue Medications

In case of symptoms of gastritis during the preparatory phase, *Laghusutasekhara rasa* will be administered. During *snehapana*, if there is nausea, vomiting, or episodes of diarrhea, the patient will be observed for stable vitals, and enough hydration will be administered using an oral rehydration solution. If any episodes of dizziness occur during *Vamana* or *Virechana*, enough hydration will be provided with a oral rehydration solution. *Ayoga* (signs and symptoms indicative of inadequate purification) or *Atiyoga* (signs and symptoms indicative of excessive purification) following *Vamana* and *Virechana* will be managed according to the standard textual practices. All such instances will be recorded in the CRF.

### Concomitant Care

The study includes only healthy volunteers; therefore, no concomitant care is needed. If any adverse events occur, they will be managed accordingly. Participants are strictly prohibited from self-administering any over-the-counter antacids, antiemetics, antidiarrheals, or conventional laxatives during the trial, as these would confound the assessment of the purification procedures. Enrolling in external physical therapies, attending commercial spa treatments, or initiating new dietary supplements outside the protocol are also restricted.

### Modifications

In this exploratory study, telomere length was initially included as one of the secondary outcome measures. The challenge with assessing telomere length was identified at the time of recruitment of the first cohort into the study. Lack of local expertise, significant logistics challenges with external testing, and prohibitive costs, all without funding, made measuring telomere length questionable. Hence, telomere length measurement was removed with approval from the institutional ethics committee (IEC), Amrita School of Ayurveda (IEC/ASA.11/27/25) on December 27, 2025. The change was updated in the CTRI (CTRI/2024/02/062176).

### Outcome Measures

#### Primary Outcome

The primary outcome is a decrease in serum MDA levels and increase in serum SOD values. A significant reduction in serum MDA values (baseline and postintervention), indicated by a *P* value <.05, along with a moderate to large Cohen *d* value suggesting a meaningful change will indicate a reduction in oxidative stress levels. A significant increase in SOD activity (baseline and postintervention), indicated by a *P* value <.05, along with a moderate to large Cohen *d* value suggesting a meaningful change reflects an enhanced biological defense mechanism and improved metabolic resilience following the therapy.

#### Secondary Outcomes

A significant increase in serum levels of telomerase activity (baseline and postintervention), indicated by a *P* value <.05, along with a moderate to large Cohen *d* value suggesting a meaningful change will be interpreted as evidence of the regenerative impact of the protocol. Higher telomerase activity is linked to increased cellular lifespan and delayed biological aging.

### Data Collection Instruments: Technical Specifications, Reliability, and Validity

Data collection is performed using standardized, high-performance commercial biochemical assays and laboratory instruments. The specific instruments, along with their established analytical reliability and validity profiles, are detailed in the following sections.

#### MDA Assay

Serum MDA is quantified as an index of lipid peroxidation using a high-sensitivity Colorimetric Assay Kit (E-BC-K025-S [16]; using the thiobarbituric acid reactive substances method). Absorbance is measured at 532 nm using a calibrated multimode microplate spectrophotometer reader. The assay exhibits high analytical validity with a linear detection range spanning from 0.1 nmol/mL to 100 nmol/mL and is sensitive to analyze 0.38 nmol/mL. The instrument setup maintains an intra-assay coefficient of variation (CV) of 4.9% and an interassay CV of 8.0%, ensuring excellent analytical reproducibility across separate experimental runs.

#### Total SOD Activity Assay

Serum total SOD (T-SOD) activity is quantitatively measured using a standardized Elabscience T-SOD Activity Assay Kit (hydroxylamine method, catalog number: E-BC-K019-S) [17]. Absorbance is measured at 550 nm using a calibrated spectrophotometer instrument. The assay uses a xanthine and xanthine oxidase reaction system to generate superoxide anion free radicals that oxidize hydroxylamine to form nitrite. Nitrite reacts with a developer to form a purplish-red compound. Endogenous T-SOD in the serum specifically intercepts and disproportionates the superoxide radicals, inhibiting nitrite formation. The resulting reduction in absorbance is perfectly, negatively correlated with serum T-SOD activity, ensuring high analytical validity and specificity for biological SOD isoforms.

#### Analytical Ranges

The instrument setup delivers a certified sensitivity limit of 4.7 U/mL and maintains a broad, clinically validated linear detection range of 4.7‐166 U/mL.

#### Reliability (Precision)

The kit protocol uses internal controls, double-distilled water blanks, and strict room-temperature equilibration routines to guarantee reproducibility. Run reliability is reinforced by performing sample evaluations in duplicate to maintain a strict intra-assay precision CV threshold of 2.8% and an inter-assay precision CV of 6.3%.

#### Telomerase Activity Assay

Quantitative determination of human telomerase concentration is executed using a standardized commercial Elabscience Human TE ELISA Kit (sandwich-ELISA method, catalog number: E-EL-H0164) [18]. Optical density is measured spectrophotometrically at a wavelength of λ=450 nm±2 nm using a calibrated microplate reader. The instrument setup uses a precoated microplate with an antibody specific to human telomerase. Following sample incubation, a biotinylated detection antibody specific for human telomerase is added. Substrate reaction is colorimetrically recorded via progressive blue-to-yellow conversion following the addition of an acidic stop solution. The method provides excellent analytical validity with zero cross-reactivity or interference from structural analogs. The instrument setup features an exact analytical sensitivity limit of 0.1 ng/mL

#### Analytical Ranges

The assay delivers an exact sensitivity limit of 0.09 ng/mL and tracks a linear detection range of 0.16‐10 ng/mL.

### Data Quality Assurance Processes

To safeguard the accuracy and integrity of the clinical dataset, data quality is strictly managed through triplicate testing and blinded sample coding. To eliminate analytical errors, serum samples for MDA, T-SOD, and human telomerase are analyzed strictly in triplicate wells, with an automatic re-assay protocol enforced if the variance between duplicates exceeds a CV of 10%. Furthermore, all specimen tubes are stripped of identifiers and masked with unique alphanumeric codes, ensuring that laboratory personnel remain completely blinded to participant identities and trial time points throughout the assay phase.

### Data Collection Personnel and Process

The collection of samples is done at the hospital laboratory by trained lab technicians. The biochemical analysis is done by a trained personnel at the Amrita Centre for Advanced Research in Ayurveda lab to generate the values of MDA, SOD, and telomerase activity. The principal investigator collects the report and records them in the CRF. No data collection is performed by participants, caregivers, or nonclinical personnel.

### Core Outcome Set and Summary Measures

Currently, no standardized core outcome set has been developed or registered for the evaluation of Panchakarma procedures (such as *Snehapana, Vamana*, and *Virechana*) in healthy volunteer populations. Consequently, the biomarkers chosen for this exploratory study—MDA, SOD, and telomerase activity—were selected independently based on cellular aging and oxidative stress hypotheses.

The continuous marker of lipid peroxidation (serum MDA levels), biological indicator of antioxidant defense (serum SOD enzyme activity values), and physiological marker of cellular longevity (telomerase activity) will be reported using the mean change from baseline to postintervention (along with the SD and 95% CIs) calculated for the study group. In addition to these mean changes, the proportion of participants experiencing any incidental rescue events (such as the percentage of volunteers) will be tracked descriptively as feasibility and safety outcomes.

### Data Collection Form Accessibility

This protocol covers an ongoing exploratory trial with data collection currently in progress; the data logging layout is fully integrated within the study’s clinical CRF. This form includes dedicated fields for tracking participant alphanumeric codes, specimen collection time stamps, and laboratory assay results (MDA, T-SOD, and telomerase concentrations). The blank data collection templates within the CRF framework are securely archived and can be provided by the corresponding author to reviewers or editors upon reasonable academic requests.

### Participant Retention and Discontinuation Protocols

Participant retention is optimized through the fully managed inpatient environment. This trial tracks an existing clinical service where healthy volunteers are admitted as full hospital inpatients for the duration of the *Śodhana* therapy; therefore, typical outpatient compliance barriers like travel issues, appointments being forgotten, or accidental dietary deviations are naturally eliminated.

For participants who voluntarily withdraw or deviate from the intervention protocols after formal enrollment, active procedures are immediately halted to protect participant autonomy and safety. Unless written consent is completely revoked, the specific outcome data collected and retained for these individuals will consist of baseline serum MDA, T-SOD, and telomerase levels, along with a documented record of the exact chronological point and clinical reason for the protocol deviation or study exit. The reasons for nonadherence and nonretention will be recorded on the CRF. All recorded reasons will be compiled into a standardized trial flow diagram and fully disclosed in the final manuscript publication.

### Criteria for the Termination of the Trial

The premature or scheduled termination of the trial will be executed immediately by the principal investigators, in absolute coordination with the IEC upon meeting any of the predefined criteria described in the following sections.

#### Expected Completion

The trial will be naturally terminated once the targeted sample size of 53 healthy volunteers has successfully completed the *Śodhana* protocol along with all corresponding baseline and postintervention laboratory evaluations.

#### Early Statistical Endpoints

The study may be halted prematurely if an early boundary condition is met, such as reaching a definitive positive or negative statistical endpoint sooner than anticipated during final data monitoring.

#### Serious Adverse Reactions

The trial will be shut down immediately if any participant experiences serious adverse reactions, including severe abnormalities in vital signs (eg, severe cardiovascular instability), persistent biological distress, or unexpected toxicological outcomes.

#### Futility Determination

The study will be terminated early if a midpoint clinical review or administrative audit leads to a clear determination that no statistically significant or clinically viable result can be obtained due to structural protocol constraints or unanticipated sample anomalies.

### Sample Size

The sample size is feasibility-driven. It was chosen based on pragmatic constraints. As this is an exploratory study, the goal is not to achieve definitive statistical power but to establish a foundation for future, larger-scale confirmatory research. Analyzing the census of Kerala, only 30% of the population could be sampled as healthy volunteers excluding NCDs, infectious diseases, other systemic illness like cancer, and age. The sample size was calculated as 53 using the following formula;


$$n=\frac{Z_{1-\alpha /2}^{2}\times P(1-P)}{d^{2}}$$

where Z_1-α/2_=1.96 (assuming a 95% CI), *P*=.5 (expected effeciency/anticipated proportion), and d=0.15 (15% margin of error), assuming a dropout rate of 20%.

Although this sample size is rooted in regional screening feasibility, a retrospective sensitivity power analysis confirmed that a finalized cohort of 53 individuals provides 80% statistical power (*α*=.05) to detect a small-to-medium intra-individual effect size (Cohen *d*=0.395) using a 2-sided paired samples framework. This standard biostatistical validation confirmed that, although the enrollment target is grounded in regional fieldwork constraints, it remains mathematically sensitive enough to capture subtle, homeostatic optimization trends in continuous aging biomarkers (MDA, SOD, and telomerase activity) within a healthy cohort.

### Recruitment

Administration of *Vasanta Vamana* and *Sarad Virechana* has been a years-long practice in our institute. The required sample will be recruited from the target population in line with the inclusion and exclusion criteria of the study.

### Ethical Considerations

#### Study Approval

This study protocol was reviewed and approved by the IEC, Amrita School of Ayurveda (approval number: IEC.ASA.PHDR.02).

#### Patient and Public Involvement

This study does not involve patients nor the public in the conceptualization, design, active conduct, or reporting stages of the trial. The entire project framework, choice of laboratory endpoints, and operational scheduling were independently developed and finalized by the investigator team. The active clinical study is being conducted exclusively within our institutional hospital under the direct supervision of the investigators, with participants involved solely as healthy clinical trial volunteers.

#### Informed Consent Procedure, Data Collection, and Documentation

*Vasantha Vamana* and *Sarad Virechana* have been standard clinical practice at our institute for many years. Individuals who are interested in personal health maintenance voluntarily register for these seasonal purificatory sessions completely independent of the research. Provided the registered person is a healthy volunteer, the person is formally invited to join the research study. Written informed consent is obtained from participants before inclusion in the study. It explicitly states that participation is entirely voluntary and that they retain the right to freely withdraw from the study at any point without any clinical penalty or compromise to their ongoing care.

Consent for participation in the study is documented in the informed consent form attached to a comprehensive patient information sheet (PIS), which completely details the procedures, potential risks, and benefits of participating in the study. Participants will be given ample time to thoroughly review the PIS and ask questions before signing the informed consent form.

The process of disclosing study parameters, answering participant questions, and obtaining formal written informed consent is executed exclusively by the principal investigator of the trial. The principal investigator is a qualified Ayurvedic physician holding a Doctor of Medicine in Ayurveda (MD-Ayur) degree with more than 10 years of active institutional clinical and research experience. Furthermore, the principal investigator has undergone formal Good Clinical Practice (GCP) training and research ethics certification. This guarantees that the consent process is completely transparent, noncoercive, and conducted in strict compliance with global bioethical standards and clinical trial regulations.

The informed consent forms will be safely stored as hard copies with limited access by the investigator team including the PhD scholar (principal investigator), guide (co-investigator), and co-guide (co-investigator). A detailed historical and clinical evalutaion along with the baseline data of the volunteers will be documented in the CRF. Each CRF has a unique ID and is anonymized. The data will be kept confidential and accessible only to the aforementioned team.

#### Data and Materials Sharing Statement

The protocol covers an ongoing exploratory trial embedded within a specific institutional clinical framework. No secondary sharing of individual participant data, de-identified biochemical metrics, or data dictionaries with external parties is planned. All raw laboratory values for serum MDA, T-SOD, and human telomerase will be securely archived and restricted exclusively to the primary institutional trial investigators to maintain strict data custody and protect participant privacy. The baseline and postintervention statistical outcomes will be fully disclosed through peer-reviewed journal publications, but the raw underlying patient-level datasets will not be deposited into public repositories or transferred to external users. Since no secondary data sharing is permitted under this study design, no application processes, transfer pipelines, or secondary consent clauses are applicable to this protocol. *Vamana* and *Virechana* follow classic, standardized institutional clinical protocols; therefore, no proprietary participant handbooks or training videos are used in this trial.

#### Safety

Adverse events, if any, occurring during the trial that are observed by the investigator or reported by the participant will be recorded on the CRF whether or not it is attributed to the trial medication. We will record the following information: description, date of onset and end date, severity, assessment of relatedness to trial medication, other suspect drug or device, and action taken. Considering the temporal relationship with the study drug/intervention and clinical presentation, causality will be independently evaluated by the principal investigator and co-investigator. All adverse events will be followed until resolution or stabilization.

#### Data Monitoring and Oversight Framework

This protocol is conducted as a single-center, investigator-initiated doctoral (PhD) research study in which the primary PhD scholar serves directly as the principal investigator. A separate, independent Data Monitoring Committee (DMC) is not required nor utilized. The trial evaluates standardized, time-tested institutional clinical procedures (*Vamana* and *Virechana*) that possess an established clinical safety track record, introducing minimal risk to participants. Instead of an external DMC, comprehensive scientific and data monitoring is executed directly by the candidate’s Doctoral Thesis Advisory Committee, which provides continuous academic and methodological oversight. Furthermore, participant safety monitoring, adverse event tracking, and ethical compliance are supervised directly by the IEC through scheduled periodic progress reports, with day-to-day data verification managed closely by the PhD scholar in her capacity as the principal investigator.

#### Trial Monitoring Processes

This protocol operates as a single-center, investigator-initiated doctoral research study. There are no external third parties nor independent clinical trial monitoring agencies involved in operational auditing. Instead, trial conduct, data entry, and study progress are monitored internally through a structured, dual-tiered academic tracking framework. First, the PhD scholar reports directly to the supervising research guide every month to review day-to-day trial operations, evaluate recruitment milestones, and cross-check master CRF data entries against raw laboratory sheets. Second, formal biannual Doctoral Committee meetings are held every 6 months to comprehensively monitor and audit overall trial progress, verify 100% of signed informed consent records, and ensure strict compliance with protocol safety guidelines and ethical frameworks. The verified monitoring updates are systematically maintained and filed with the IEC during routine reporting windows to guarantee data integrity.

#### Risk-Benefit Balance, Compensation, and Adverse Event Management

Since *sodhana* procedures require temporary modification of diet and lifestyle, to avoid any medical concerns, the participants are kept as inpatients under the supervision of the investigators and trained medical staff throughout the study. As a direct benefit of participation, all diagnostic screenings, including hematology, renal, hepatic, and thyroid panels, will be conducted entirely free of cost. In the highly unlikely event that any adverse event occurs during the trial period, it will be immediately and comprehensively managed medically by the institute at no expense to the participant.

#### Ancillary and Posttrial Care Provisions

Although this trial evaluates safe, standardized clinical protocols with healthy volunteers, full ancillary care is guaranteed throughout the inpatient stay. If a participant develops any routine, non-trial-related acute condition while hospitalized, complete medical management, prescriptions, and consultations are provided onsite entirely free of cost through the institute’s standard clinical infrastructure.

*Saṃsarjana Krama* is structurally embedded as part of the active study intervention. Posttrial care begins immediately following the final postintervention blood draw. Upon protocol completion and official discharge, a direct telephonic support gateway is established for the subsequent 30 days. This gives participants free access to the investigator team and institutional outpatient physicians for any health-related guidance, safety reporting, or routine medical consultations to ensure a smooth transition back to their standard daily routines.

#### Confidentiality and Data Privacy Security

To safeguard participant privacy and comply with biomedical ethics regulations, a strict confidentiality protocol is enforced throughout all phases of the study. Before the trial, personal information collected during the initial registration and screening phase is kept confidential within the hospital’s secure registration logs. During the trial, all enrolled participants are immediately assigned a unique alphanumeric identification code. All subsequent records, CRFs, and laboratory blood collection tubes are labeled exclusively with this code; no names, hospital file numbers, or identifying demographics are attached to biological samples or data entry sheets. Physical documents including signed informed consent forms and raw laboratory data sheets are stored in a locked filing cabinet accessible solely by the principal investigator. Digital datasets are maintained on a password-protected, encrypted local storage drive with no external network connectivity. After the trial, the fully anonymized data will be kept securely for a mandatory archival period of 5 years before permanent deletion, and no identifying personal details will ever be shared with external parties or disclosed in peer-reviewed publications.

### Statistical Measures

The change in values of the outcome measures including MDA, SOD, telomerase activity, and telomere length will be recorded. The data will be tested for normality using the Shapiro-Wilk test. They will be tested for significance using paired *t* tests (if the data are normally distributed) or the Wilcoxon Signed Rank test (if the data are not normally distributed). A *P* value <.05 will be considered significant. All statistical analysis will be conducted using SPSS version 31 (IBM Corp). Moreover, a secondary exploratory subgroup analysis will be conducted within and between the 2 cohorts (*Vamana* and *Virechana* groups) based on the distribution of the data. If the data are normally distributed, within-group analysis will be conducted using paired *t* tests and between-group analysis will be conducted with *t* tests, whereas Wilcoxon Sign Ranked tests and Mann-Whitney *U* tests will be used, respectively, for data that are not normally distributed.

Cohen *d* effect sizes will also be calculated to address the exploratory nature of the study and evaluate the clinical and biological relevance of changes in biomarkers (ie, MDA, SOD, and telomerase activity). Results will be interpreted based on the magnitude and directionality of these effect sizes (small: 0.2; medium: 0.5; large: 0.8) to provide a standardized measure of treatment impact beyond simple *P* value testing.

Given the exploratory, hypothesis-generating design of this pilot study, secondary multivariable analyses will be introduced to evaluate the influence of potential baseline confounders. For normally distributed continuous data, an analysis of covariance (ANCOVA) will compare postintervention outcomes between the 2 seasonal subcohorts (*Vamana* vs *Virechana*) using individual pre-intervention scores as the baseline covariate to mathematically adjust for initial biological variation. Additionally, to evaluate the entire cohort of 53 participants as a unified group, multiple linear regression models will be constructed using the postintervention scores as the dependent variable, entering baseline biomarker values, age, sex, and baseline BMI as independent predictors to systematically control for demographic and physical confounding variables.

### Statistical Handling of Missing Data

This trial evaluates objective biochemical endpoints within a strictly monitored hospital inpatient setting; the incidence of missing data is anticipated to be extremely low (<5%). Any missing data points are assumed to follow a missing completely at random mechanism, as data omissions would stem strictly from random logistical or laboratory anomalies.

Missing data will be handled using an available case analysis (pairwise deletion) framework for the primary longitudinal comparisons, ensuring that all available valid pairs of pre- and postintervention biochemical metrics are fully utilized without fabricating data. To verify that this approach does not introduce statistical bias, a conservative baseline observation carried forward (BOCF) sensitivity analysis will be executed concurrently. Under the BOCF framework, any missing postintervention value will be replaced by the participant’s own baseline value, assuming zero therapeutic change. This dual-validation approach guarantees a highly transparent, noninflationary evaluation of the trial’s biochemical endpoints.

### Sensitivity Analyses

A formal sensitivity analysis will be performed to address potential protocol deviations and data completeness variations. The primary longitudinal analysis (pairwise deletion tracking available cases) will be cross-verified against a conservative BOCF model. In this sensitivity check, any missing or protocol-deviated postintervention slot will be replaced directly with that participant’s baseline value, representing an assumption of zero therapeutic efficacy. The *P* values from both the primary available-case model and the secondary BOCF imputation model will be compared; if the statistical significance (*P*<.05) remains consistent across both frameworks, the biological findings of the trial will be considered methodologically stable and free from attrition bias.

### Dissemination Policy

On completion of the trial and data analysis, we will ensure that the evidence gathered reaches health care professionals and scientific community through all possible ways. We will make sure that the same information reaches the public in appropriate ways to be incorporated as a preventive strategy for age-related changes and improving the health span. The study results will be submitted to peer-reviewed journals for publication regardless of the findings. Moreover, the results will be presented at relevant academic and scientific conferences. The CTRI page will also be updated accordingly upon completion of the study.

## Results

The study started recruiting participants in March 2025, and, as of April 4, 2025, 27 participants had voluntarily participated in *Vasantha Vamana*. The data analysis is pending. We expect to reach the targeted number of participants by December 2026. The data analysis will be conducted, and the results will be submitted for publication within the 6 months following study completion.

## Discussion

### Theories Underpinning the Study

*Ritu sodhana* or seasonal purification is generally intended to eliminate the seasonal accumulation of humors. This process thereby reduces the susceptibility of seasonal disorders [14]. Acharya Charaka in the context of *Ritu sodhana* has put forth an additional statement that administration of seasonal biopurification will bestow rejuvenation and improve health span [19]. Even though these procedures have been evaluated for their clinical efficacies, its influence on biomarkers for aging has not yet been evaluated. This gap underpins the development of this research protocol, which aims to generate preliminary evidence for this purpose.

*Trikatu Churna,* used for *pachana* and *deepana*, has bioactive constituents including piperine, gingerols, and shogaols and has demonstrated effects on gastric secretion, gut motility, enzymatic activity, splanchnic circulation, and nutrient bioavailability, supporting improved digestive efficiency and systemic absorption [20,21]. This serves as metabolic priming, which is essential to ensure effective digestion and absorption prior to the administration of ghee. *Moorchita ghruta* [22] is widely used in clinical practice. Since the cohort includes healthy volunteers, no other medicated ghee was chosen. *Moorchita taila* [23] was chosen for external application owing to its popularity for the panchakarma procedure. *Trivrut lehya* is used for *Virechanakarma* with limited complications [24].

Based on this framework, we expect that *sodhana* will aid with releasing the waste metabolites, thereby influencing the values of MDA, SOD, and telomerase activity to trigger measurable anti-aging pathway responses across our cohort of 53 healthy volunteers. The hypothesized primary findings include a statistically significant pre-to-postintervention drop in oxidative tissue damage evidenced by a reduction in MDA levels coupled with a significant upregulation of cellular antioxidant defenses, marked by enhanced SOD activity. Furthermore, it is anticipated that this intensive detoxification phase will positively modulate cellular longevity clocks, manifesting as stabilized or elevated telomerase activity, thereby providing an objective, molecular validation of Acharya Charaka’s classical rejuvenation hypothesis.

There are multiple theories supporting the process of aging. However, evidence for the molecular, cellular, or physiological changes involved in the process of aging and the associations among them is lacking [25]. Oxidative damage is one theory for aging. The multiple interrelated reactions involved in metabolism generate reactive oxygen species (ROS) in the body. The hypothesis that ROS accelerate the aging process is supported by genetic studies of transgenic animals engineered to overexpress or lack antioxidant enzymes. A study concluded that the life span of *Drosophila melanogaster* is extended by the overexpression of antioxidants including SOD and catalase [26]. MDA is also a widely used biomarker indicating lipid peroxidation, yet another oxidative degradation phenomenon enhancing aging. However, MDA is a stable end-product of lipid peroxidation. Therefore, it can be considered a reliable and noninvasive indicator of cumulative oxidative damage to cell membranes. SOD provides a real-time view of the body’s redox state. Unlike other aging biomarkers, it responds dynamically to changes in oxidative load [27].

Biomarkers including MDA and SOD are intended as short-term surrogate indicators of oxidative stress. They are not direct measures of aging. In a sample of 43 relatively healthy individuals, a 21-day fasting period influenced the levels of blood oxidative stress biomarkers including MDA [28]. In addition, a 30-day, high-intensity, interval running training course proved to be more effective at reducing MDA levels and increasing SOD levels in young men [29]. These existing data demonstrate that a 21-day to 30-day intervention window is physiologically sufficient to induce significant, measurable alterations in human blood oxidative stress parameters. Consequently, these established timelines provide strong chronological justification for the 21-day duration of our intensive inpatient *Śodhana* protocol, confirming that our study window is fully optimized to capture variations in the biomarkers.

Due to end-replication problems, telomeres are shortened after each cell division. As such, telomere length is regarded a marker of aging. Telomere integrity largely depends on telomerase activity [30]. Therefore, enhancing telomerase activity is the target of many therapeutic approaches aiming to maintain telomere length and thereby by regulating the cellular heath span. The telomerase enzyme counteracts telomere attrition by catalyzing the addition of TTAGGG nucleotides to the chromosome end [30,31].

Incorporating variables like MDA, SOD, and telomerase activity enable us to strengthen the theories documented in traditional texts by generating hypotheses based on scientific evidence. This study will serve as a feasibility trial, generating data that could inform larger clinical trials.

### Limitations

Placebos for procedures like *Vamana* and *Virechana* are challenging to include; therefore, this study does not include a control group. However, the resulting single-group study design raises concerns about internal validity.

In addition, this exploratory pilot trial lacks a concurrent, untreated parallel control group. Since this study operates as an investigator-initiated doctoral project embedded within a real-world clinical service, introducing an untreated healthy cohort was operationally and ethically unfeasible, as all recruited individuals are actively seeking immediate wellness interventions. To mitigate this constraint and maximize data validity, each participant serves as their own internal longitudinal control through structured pre- and postintervention paired comparisons. Furthermore, potential lifestyle and environmental confounding variables are strictly controlled by housing all 53 participants within an identical inpatient ward environment throughout the active study timeline.

We acknowledge that serum MDA and T-SOD are short-term surrogate markers of oxidative stress, not direct proof of delayed aging. In this protocol, *Śodhana* represents a complete therapeutic process including *Pūrvakarma* (internal oleation), *Pradhāna Karma* (*Vamana* or *Virechana*), and *Saṃsarjana Krama*. Therefore, acute changes in MDA and T-SOD reflect the immediate metabolic shifts of this entire multiphase cycle rather than long-term aging changes. To address this limitation, we paired these dynamic redox markers with telomerase activity. This allows us to explore whether the intense physiological “reset” caused by the complete *Śodhana* sequence correlates with any stabilizing effects on a deeper, genetic marker of cellular longevity.

The sample size calculation was also pragmatic rather than inferential. The exploratory nature of the study warrants a focus on feasibility of the protocol rather than to test the hypothesis.

The recruitment is seasonal and consecutive. Therefore, differences in environmental factors, including the climate and diet, as well as baseline biological rhythms between the 2 cohorts may act as potential modifiers for biomarker levels.

Although other biomarkers, including an interleukin panel and the microbiome, are available, we included only a limited number of biomarkers for our healthy volunteers due to financial constraints.

Regardless of these limitations, the study will generate preliminary evidence of how these procedures can affect oxidative stress levels. This could facilitate introducing *Vamana* and *Virechana* as preventive procedures in additional medical arenas other than as a preventive aging intervention. The data regarding change in the telomerase activity and telomere length will provide a new avenue for further studies.

### Future Directions

As an exploratory and hypothesis-generating pilot study, the definitive dataset generated from these 53 participants will establish the baseline standard deviations and accurate effect sizes required to design future research. The immediate next phase of this work will involve developing a fully powered, multi-arm, randomized controlled trial. This future trial will introduce a parallel, nontreatment control group or a fasting-mimicking dietary control arm, which is necessary to cleanly separate the unique cellular detoxification effects of active *Śodhana* from simple caloric restriction.

## Abbreviations

ANCOVA: analysis of covariance
BOCF: baseline observation carried forward
CBC: complete blood count
CRF: case report form
CTRI: Clinical Trial Registry-India
CV: coefficient of variation
DMC: Data Monitoring Committee
GCP: Good Clinical Practice
ICH: International Conference on Harmonisation
IEC: institutional ethics committee
MDA: malondialdehyde
NCD: noncommunicable disease
PIS: patient information sheet
ROS: reactive oxygen species
SOD: superoxide dismutase
SOP: standard operating procedure
T-SOD: total superoxide dismutase
WHO: World Health Organization
